# Bilateral *Candida* keratitis in an HIV patient with asymptomatic genitourinary candidiasis in Uganda

**DOI:** 10.1016/j.mmcr.2018.07.007

**Published:** 2018-07-17

**Authors:** Simon Arunga, Teddy Kwaga, Astrid Leck, Victor H. Hu, Matthew J. Burton

**Affiliations:** a Department of Ophthalmology, Mbarara University of Science and Technology, P.O Box 1410, Mbarara, Uganda; b International Centre for Eye Health, London School of Hygiene & Tropical Medicine, London, UK

**Keywords:** Fungal keratitis, Candida keratitis, Genitourinary candidiasis, HIV, Uganda

## Abstract

A 35-year-old male presented with Candida keratitis in the left eye. He was HIV positive with a CD4 of 352 cells/µL. The eye quickly deteriorated, despite intensive antifungal treatment and was eviscerated. Five months later, he re-presented with Candida keratitis in his right eye. A focal source of Candida infection was suspected and a urine culture identified Candida spp, despite being asymptomatic for genitourinary candidiasis. He was subsequently treated with good outcome (max. 75 words)

## 1 Introduction

Microbial keratitis (MK) is caused by a range of pathogens, including bacteria, viruses, protozoa and fungi. It is characterized by pain, conjunctival hyperemia and corneal ulceration with stromal inflammatory cell infiltrate. MK frequently leads to sight-loss from dense corneal scarring or even loss of the eye when severe.

In tropical regions approximately half of MK is attributable to fungal pathogens [1], [2]. Filamentous organisms predominate, with *Fusarium spp*. and *Aspergillus spp*. accounting for the large majority [3]. Yeast infections, mostly caused by *Candida spp* are less frequent. In contrast, in temperate regions yeast often predominate, although some recent reports suggest an increasing proportion of filamentous infections [4]. Reported risk factors for fungal keratitis include trauma, ocular surface disease, contact lens use, prior surgery, traditional eye medicine (TEM), steroid use and immunosuppression [4], [5], [6].

*Candida* keratitis is particularly associated with chronic ocular surface disease and has been reported following various corneal procedures [4], [7], [8]. Although the source of the *Candida* is usually exogenous, it may sometimes have an endogenous source such as from the oral and genitourinary surfaces or a disseminated systemic infection in severely immunocompromised individuals [9], [10]. Genitourinary *Candida* infection is relatively common in Africa; it can be either symptomatic or asymptomatic [11]. It is reported to contribute 30–50% of all cases treated with genitourinary infection [11], [12], [13], [14]. However, it has not been previously reported to be associated with keratitis.

Here we report a case of a 35-year-old man with sequential bilateral *Candida* keratitis with a concomitant asymptomatic genitourinary *Candida* infection. This provides important lessons on investigation, treatment and preventative care in similar cases.

## 2 Case

### 2.1 First eye presentation

A 35-year-old male Ugandan presented to Mbarara University Referral Hospital Eye Centre (MURHEC) in June 2017 with a 10-day history of a painful, red left eye. There was no history of trauma, contact lens or TEM use. He was not aware of his HIV status at the time of presentation, but thought that he was HIV negative. He described a somewhat similar eye problem in his teenage years, which followed trauma, was treated and had healed. He had experienced no further ocular problems until this new presentation.

**Fig. 1:**
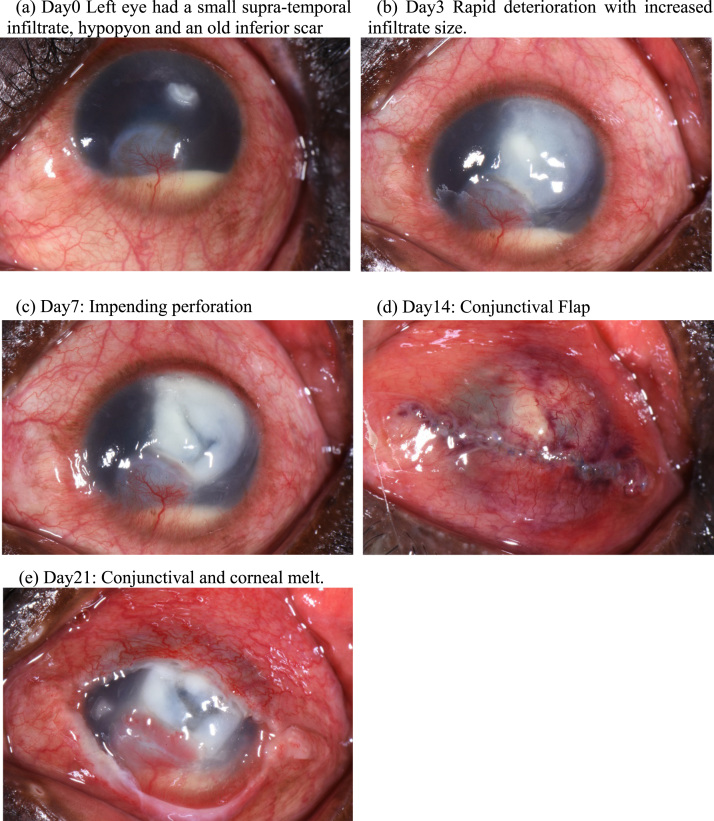
(a–e) showing appearance of the left eye from presentation (day0) to day21, in June 2017.

On this admission (day0),the left visual acuity was hand movements only, with no improvement on pinhole. There was a dense white paraxial supratemporal corneal infiltrate (2.0 mm × 1.5 mm), an overlying epithelial defect (2.0 mm × 1.5 mm), 80% corneal thinning and a 3.5 mm hypopyon (Fig. 1a). Additionally, the left cornea had an old inferior vascularized scar (7 mm × 6 mm). The right eye had an unaided visual acuity of 6/5 and normal ocular examination.

Corneal scrapings were collected for microscopy (Gram stain, Potassium Hydroxide [KOH] stain, Calcofluor White [CFW] stain, Lactophenol Cotton Blue stain[LPCB]) and culture (Blood Agar [BA], Chocolate Agar [CA], Potato Dextrose Agar [PDA] and Brain Heart Infusion [BHI]). Initial CFW slide revealed fungal elements. The Gram, KOH and LPCB tests were negative. However, *Candida spp*. grew on BA, PDA, CA and BHI subculture within 48 h.

The patient was started on hourly Natamycin 5% eyedrops (Zonat Sunways India) as well as Ofloxacin 0.3% eyedrops (Biomedica Remedies-India) 4 times/day and Atropine eyedrops. By day3, the eye had rapidly deteriorated (Fig. 1b) and hourly Chlorohexidine 0.2% eyedrops (locally formulated) was added to his treatment. By day7 the cornea had thinned further and was threatening to perforate (Fig. 1c). Corneal tissue for transplantation is currently unavailable in Uganda. On day8, a conjunctival flap procedure was performed (Fig. 1d), in conjunction with a subconjunctival injection of Fluconazole 2% (0.5 ml). On day21, he returned with a total corneal and conjunctival flap melt (Fig. 1e). At this stage further active treatment was considered futile and a decision was taken with the patient to perform an evisceration. Subsequently, a prosthetic shell was fitted.

It is our routine practice to offer HIV counselling and testing to all people presenting with MK. This individual accepted the offer and was found to be HIV positive. He was referred to HIV services and started anti-retroviral therapy. His CD4 count was 352 cells/µL around the time treatment was initiated.

### 2.2 Second eye presentation

**Fig. 2:**
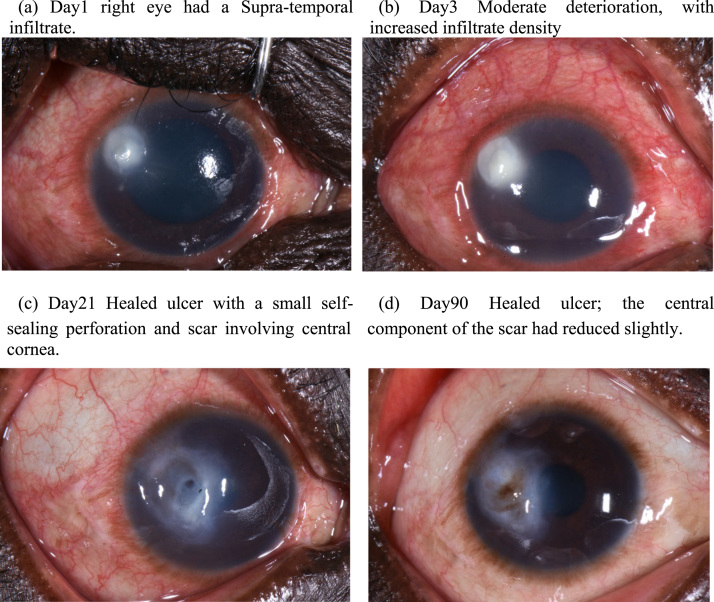
(a–d) showing appearance of the right eye from presentation (day0) to day90, starting in December 2017.

Five months later, he returned to MURHEC with a 4 day history of a painful right eye. Again, there was no history of trauma, contact lens or TEM use. On this day0 for the righteye presentation, visual acuity in the right eye was 6/12. Slit lamp examination showed a supra-temporal dense corneal infiltrate (3.1 mm × 2.8 mm), Fig. 2a. Corneal scrape samples were collected and sent for microbiological investigations, as outlined above. Gram stain showed pseudo-hyphae. CFW and KOH reported fungal hyphae and all culture plates (BHI subculture, BA, CA, PDA) grew *Candida spp*. The same first line protocol as previous (Natamycin, Ofloxacin and Atropine) was started. At this point, we were concerned that he might have a source of *Candida* elsewhere, that had led to the sequential corneal infections. He reported no systemic symptoms; specifically he did not have dysuria. As part of the assessment a urine sample was cultured, which also grew *Candida spp*.

By day3 we noted a moderate deterioration (Fig. 2b). Therefore, we added hourly Amphotericin B 0.15% eyedrops (locally formulated with a hyper methylcellolose base) and oral fluconazole 200 mg twice a day to his treatment. By day21, the ocular pain had greatly reduced and the infiltrate had transitioned into a scar extending to the visual axis (7 mm × 4 mm). He developed a small para-central perforation. This self-sealed with iris plugging; the anterior chamber was deep and Siedel's test was negative (Fig. 2c). By 3 months (day90) the scar size had reduced slightly (6 mm × 3.2 mm), and his right visual acuity was 6/24.

## 3 Discussion

Although *Candida* keratitis has generally been found to be more common in temperate climates, it has been reported, albeit less frequently, in tropical regions [1], [2], [15]. This patient presented us a unique opportunity to reflect on the presentation of *Candida* keratitis in HIV infected patients and identify key considerations to ensure a good outcome.

Firstly, the patient had undiagnosed HIV infection with a relatively low CD4 count which could have predisposed him to the initial infection. We routinely provide HIV counselling and testing to MK patients as part of our hospital protocol, based on previous studies in the region that noted a high proportion of MK patients with HIV [16], [17]. This is consistent with our experience in Uganda, where we find in ongoing case-control work HIV is more frequent in people with MK (unpublished data).

Secondly, we did not initially suspect a systemic source of the *Candida* infection. The patient was asymptomatic for this. Endophthalmitis resulting from *Candida* septicemia is well characterized [10], [18], [19]. Blood culture for *Candida* septicemia was not performed in our patient because he was afebrile, he did not have oral thrush and otherwise clinically well. Patients with *Candida* septicemia are usually very sick at presentation; they require hospitalization, with a majority requiring intensive care treatment [18]. Our patient was found to have asymptomatic genitourinary candidiasis on urine culture. Therefore, we think that the most likely explanation for the acquisition of his sequential case bilateral *Candida* keratitis was due to poor hygiene.

Thirdly, our patient rapidly deteriorated on the first presentation resulting loss of the eye, despite intensive treatment with two anti-fungal agents. *Candida* keratitis rapidly causes corneal perforations, corneal scars, endophthalmitis and loss of vision in many cases [20]. However, experience of managing his first infection helped us to aggressively manage his remaining eye when it became infected. Prompt microbiological confirmation of the *Candida* helped to initiate a dual drug combination of hourly Natamycin 5% eyedrops and Amphotericin B 0.15% eyedrops. We were able to save the eye and preserve useful vision. Molecular strain typing which would be required to validate if there was any similarity among the isolates, was not available.

This case graphically illustrates the increased risk to fungal keratitis experienced by HIV positive individuals. It highlights the need in unusual bilateral cases for careful assessment for a potential source elsewhere in the body. It is a reminder of the high ocular morbidity associated with these types of infections and the particular treatment challenges they present

## References

[1] Leck A., Thomas P., Hagan M., Kaliamurthy J., Ackuaku E., John M. (2002). Aetiology of suppurative corneal ulcers in Ghana and south India, and epidemiology of fungal keratitis. Br. J. Ophthalmol..

[2] Hagan M., Wright E., Newman M., Dolin P., Johnson G. (1995). Causes of suppurative keratitis in Ghana. Br. J. Ophthalmol..

[3] Srinivasan M. (2004). Fungal keratitis. Curr. Opin. Ophthalmol..

[4] Ong H.S., Fung S.S., Macleod D., Dart J.K., Tuft S.J., Burton M.J. (2016). Altered patterns of fungal keratitis at a London ophthalmic referral hospital: an eight-year retrospective observational study. Am. J. Ophthalmol..

[5] Bharathi M.J., Ramakrishnan R., Meenakshi R., Padmavathy S., Shivakumar C., Srinivasan M. (2007). Microbial keratitis in South India: influence of risk factors, climate, and geographical variation. Ophthalmic Epidemiol..

[6] Yorston D., Foster A. (1994). Traditional eye medicines and corneal ulceration in Tanzania. J. Trop. Med. Hyg..

[7] Sun R.L., Jones D.B., Wilhelmus K.R. (2007). Clinical characteristics and outcome of Candida keratitis. Am. J. Ophthalmol..

[8] Thomas P., Kaliamurthy J. (2013). Mycotic keratitis: epidemiology, diagnosis and management. Clin. Microbiol. Infect..

[9] S. Motukupally, V. Nanapur, K. Chathoth, S. Murthy, R. Pappuru, A. Mallick, et al. Ocular infections caused by Candida species: Type of species, in vitro susceptibility and treatment outcome, 2015. 10.4103/0255-0857.167331 26470961

[10] K W.P., Tsui E., Barbazetto I., Park L. (2017). Ocular involvement in patients with fungemia in an Urban tertiary care center. Ocul. Immunol. Inflamm..

[11] Obisesan O.J., Olowe O.A., Taiwo S.S. (2015). Phenotypic detection of genitourinary candidiasis among sexually transmitted disease clinic attendees in Ladoke Akintola University Teaching Hospital, Osogbo, Nigeria. J. Environ. Public Health.

[12] Mukasa K.J., Herbert I., Daniel A., Sserunkuma K.L., Joel B., Frederick B. (2015). Antifungal susceptibility patterns of vulvovaginal Candida species among women attending antenatal clinic at Mbarara Regional Referral Hospital, South Western Uganda. Br. Microbiol. Res. J..

[13] Okungbowa F.I., Dede A.P., Isikhuemhen O.S., Okungbowa M.O. (2006). Age and marital distributions of genitourinary candidiasis among symptomatic women in Nigeria. Med. J. Islam. World Acad. Sci..

[14] Sehgal S. (1990). Epidemiology of male urethritis in Nigeria. J. Trop. Med. Hyg..

[15] Mafwiri M., Kanyaro N., Padhan D., Sanyiwa A., Sangawe J., Kinabo N. (2013). The microbial aetiology of corneal ulceration among patients attending a tertiary referral centre in Dar es Salaam. JOECSA.

[16] Mselle J. (1999). Fungal keratitis as an indicator of HIV infection in Africa. Trop. Dr..

[17] Burton M.J., Pithuwa J., Okello E., Afwamba I., Onyango J.J., Oates F. (2011). Microbial keratitis in East Africa: why are the outcomes so poor?. Ophthalmic Epidemiol..

[18] Khalid A., Clough L.A., Symons R., Mahnken J.D., Dong L., Eid A.J. (2014). Incidence and clinical predictors of ocular candidiasis in patients with Candida fungemia. Interdiscip. Perspect. Infect. Dis..

[19] Yamamoto S., Ikeda M., Fujimoto F., Okamoto K., Wakabayashi Y., Sato T. (2018). Bilateral Candida endophthalmitis accompanying Candida lusitaniae bloodstream infection: a case report. J. Infect. Chemother..

[20] Xie L., Zhong W., Shi W., Sun S. (2006). Spectrum of fungal keratitis in north China. Ophthalmology.

